# The bio-artificial pancreas to treat type 1 diabetes: Perspectives from healthcare professionals in the Netherlands

**DOI:** 10.1016/j.jcte.2024.100372

**Published:** 2024-10-17

**Authors:** Dide de Jongh, Eline Bunnik, Behiye Ozcan, Robert Zietse, Emma Massey, VANGUARD consortium

**Affiliations:** a Erasmus MC Transplant Institute, Department of Internal Medicine, University Medical Centre Rotterdam, 3000 CA Rotterdam, the Netherlands; b Department of Medical Ethics, Philosophy and History of Medicine, Erasmus MC, University Medical Centre Rotterdam, 3000 CA Rotterdam, the Netherlands; c Department of Internal Medicine, Erasmus MC, University Medical Center Rotterdam, 3000 CA Rotterdam, the Netherlands

**Keywords:** Diabetes mellitus, Type 1, Regenerative Medicine, Bioartificial organs, Psychosocial Factors, Ethics, Qualitative research

## Abstract

**Aims:**

Healthcare professionals are relevant stakeholders because of their gatekeeper role in the clinic. This study aims to explore their perspectives on the potential future clinical implementation of the bio-artificial pancreas (BAP) for people with type 1 diabetes, and suitable target groups.

**Methods:**

Semi-structured interviews were conducted with 17 healthcare professionals, including endocrinologists, nurses, and pancreas transplant surgeons. Inclusion was stopped once data saturation was reached. The audiotaped interviews were transcribed verbatim. Qualitative content analysis using an inductive approach was conducted to develop themes within a coding framework.

**Results:**

Three main themes emerged: (1) hoped-for benefits, which included improved clinical outcomes, enhanced sense of normality, reduced mental burden for patients and their significant others, greater societal participation, and lower costs; (2) concerns, which included safety and effectiveness, inequitable access, accurate information, control over self-management, and organizational challenges; and (3) allocating the BAP during initial implementation, which included prioritizing people who lack effective treatment options, people with mental health issues, and vulnerable people.

**Conclusions:**

The results of this study are important for researchers and practitioners involved in the development of the BAP, so that they can align its design and the process of clinical implementation with healthcare professionals’ perspectives.

## Highlights

• Recipients will have more mental space to focus on other aspects in life.
• The invisibility of the BAP could enhance the sense of ‘normality’ for recipients.
• Implementing the BAP could lower public healthcare costs.
• There are concerns about safety, inequitable access and relinquishing control.

## Nomenclature

BAP: bio-artificial pancreas
CGM: continuous glucose monitor
HCL: hybrid closed loop

## Introduction

A recent advance in the field of regenerative medicine is the development of a vascularized, personalized, and transplantable hybrid beta-cell replacement therapy for people living with type 1 diabetes, known as a bioartificial pancreas (BAP). This potential regenerative medicine therapy is still experimental, so describing it involves some speculation. However, there is a body of literature detailing its key characteristics [1], [2]. In such a therapy, insulin-secreting cells derived from deceased donors, pigs, or allogenic stem cells, will be combined with genetically modified cells to safeguard the BAP against immune responses, thereby avoiding the need for immunosuppression. Additionally, modified cells may also be included to promote angiogenesis and vascularization (development of new blood vessels). However, insulin-secreting cells are vulnerable to destruction when directly implanted into the body. Therefore, the cellular components may be enclosed by encapsulation materials or supported by a *scaffold* onto which cells can be distributed [1]. The materials under investigation include synthetic and decellularized biological materials, such as hydrogel membranes. The medical device shields and secures cellular components, facilitates the insertion and removal of the product, and enhances the microenvironment to optimize the function of the BAP [3]. The first safety, tolerability, and efficacy trials of BAPs are ongoing [4], [5]. Healthcare professionals are relevant stakeholders in the clinical implementation of this new technology because of their gatekeeper role in the clinic. Although they could influence the adoption and implementation of the BAP, there is a limited understanding of diabetes healthcare professionals’ perspectives regarding this potential therapy compared to existing treatment modalities. This study aims to fill this knowledge gap.

Currently, there are two treatment modalities for managing type 1 diabetes: self-management using insulin injections or a pump (with or without device-based technologies) and islet or whole pancreas transplantation. However, both of these treatment modalities have limitations [6]. For individuals with type 1 diabetes, handling advanced device-based technologies, such as hybrid closed-loop (HCL) systems, can be challenging. When using such technology, they must frequently change the site of the system, calibrate their device, count carbohydrates, and administer boluses according to lifestyle factors [7], [8]. Effective self-management of diabetes presents an even greater challenge for individuals who lack the capacity to understand the necessary tasks or who have limited health literacy [9]. Yet transplantation is limited by the necessity of immunosuppressive medication and the significant shortage of deceased donors. Hence, there is a global need to develop novel treatment approaches for people with type 1 diabetes that could overcome the limitations of the current modalities. The BAP is one such approach. It may be able to precisely replicate the function of the native pancreas without the need for immunosuppression and self-management. However, since this potential future therapy is still experimental, technical and clinical obstacles must be overcome before the BAP can be implemented in clinical practice. When these obstacles are resolved, there may be significant psycho-social factors to consider.

The uptake and implementation of the BAP could potentially be influenced by psycho-social factors. These factors include perspectives of stakeholders (such as diabetes healthcare professionals and patients) [10], socio-demographic factors (e.g., the socioeconomic status and ethnicity of persons with type 1 diabetes) [11], [12], local culture (i.e., pro- or anti-innovation) [13], and national reimbursement policies [11], [14], [15], [16], [17]. Diabetes healthcare professionals are key stakeholders, as they help individuals with type 1 diabetes in determining the most suitable treatment in accordance with clinical guidelines. They also assess whether the individual (or the caregiver) is motivated and competent to effectively manage the therapy. Numerous studies have highlighted the impact of clinicians’ communication skills [18] and their expectations of and experience with diabetes-specific technology on its uptake and implementation [19], [20], [21], [22]. Moreover, professionals can influence continuation of a therapy by addressing adherence barriers and offering ongoing support [23], [24], [25]. Yet several studies have demonstrated that clinicians may also contribute to inequitable distribution of advanced technologies among people with type 1 diabetes, as they tend to select people based on their own preconceptions and clinical judgement rather than objective criteria [21], [22], [9], [26]. These findings underscore the central role of diabetes healthcare professionals as gatekeepers influencing uptake and implementation of novel technologies in clinical practice.

Despite this central role of healthcare professionals, their perspectives on the BAP have not yet been investigated. Therefore, this study aims to explore healthcare professionals’ perspectives on the potential of the BAP as an alternative treatment, compared to currently available therapy options for type 1 diabetes. Additionally, professionals’ perspectives on suitable target groups for the BAP during its initial implementation in the clinic are investigated. This study can contribute to responsible clinical translation and implementation of this potentially groundbreaking cutting-edge strategy of restoring euglycemia in individuals with type 1 diabetes.

## Materials and methods

### Study design

This study was a cross-sectional, multi-center interview study.

### Sampling and recruitment

We recruited professionals through purposive sampling to ensure representation from diverse multidisciplinary settings, including individuals working in academic and regional hospitals as well as in urban and rural areas. This also included selecting professionals with various roles within a diabetes care team, such as endocrinologists, transplantation surgeons, and nurses caring for adults, adolescents, and children with diabetes. Potential participants were approached via email and given verbal and written information about the study.

### Data collection

Semi-structured interviews were conducted between December 2022 and May 2023 by DJ. Before the start of the interview study, an interview guide (see Supplementary Material 1) was developed by DJ, reviewed by EM, EMB, and BO (respectively, psychologist, medical ethicist, and endocrinologist), and discussed with the principal investigator of the VANGUARD consortium. This interview guide was used to structure and standardize data collection and ensure that the study aims were achieved. The researcher gave a brief standardized PowerPoint presentation providing information about the BAP at the start of the interview (for the translated script of the presentation, **see** Supplementary Material 2). All interviews lasted approximately 45 min and were audiotaped and transcribed verbatim, with assistance from the transcription program Amberscript. The transcripts were anonymized by assigning a unique number and removing all identifying information. Additionally, socio-demographic characteristics (e.g., profession, years of experience in the field of diabetes, age, and sex) were collected using a brief questionnaire. Our methods are reported in accordance with the consolidated criteria for reporting qualitative research (COREQ) [27].

### Data analysis

An inductive approach based on the general principles of grounded theory research was used [28]. One researcher (DJ) read all the transcribed interviews to record emerging ideas, then coded the transcripts line-by-line, supported by NVivo 12 software (QSR International Pty Ltd.). A coding framework is developed for this study. Two senior researchers (EM and EB) with experience in qualitative research independently coded four transcripts to refine the initial coding framework. Discrepancies in coding between DJ, EM, and EB were discussed until agreement was reached. The coded data were then thematically analyzed to identify the key themes and subthemes. DJ, EM, and EB evaluated the coding framework bi-weekly. This process was repeated until the final coding framework was agreed upon. After analyzing 14 interview transcripts, no new codes were added to the coding framework. Three more interviews were conducted and analyzed to verify that data saturation had been reached. To illustrate the themes and subthemes, representative quotations were chosen and translated into English.

### Ethical approval

The research proposal was submitted for review by the Research Ethics Review Committee of Erasmus MC and a waiver was granted (MEC-2022–0568), as the study did not fall within the scope of the Dutch Medical Research Involving Human Subjects Act (WMO). Written informed consent was obtained from all the professionals.

## Results

A total of seventeen professionals working in a diabetes care team were included in the sample. Professionals worked in academic (53 %) and regional (47 %) hospitals across rural and urban areas. Most interviews were conducted in person at the hospital (53 %), and some were conducted online depending on the professional’s preference, see Table 1 for further details. We identified three key themes and fifteen subthemes.

**Table 1:** Participants characteristics.

Characteristics	N	%
Total number of interviewees	17	100
Sex		
Female	12	70.5
Male	5	29.5
Hospital		
Academic	9	42.9
Regional	8	47.1
Profession		
Endocrinologists	8	47.1
Pancreas transplant surgeons	2	11.8
Nurses	7	41.2
Experience of working in diabetes care (years)		
<5	2	11.8
5–10	3	17.6
>10	12	70.6

### Theme 1: Hoped-for benefits of the BAP for recipients and society

Professionals expected various benefits that the BAP could offer to people with type 1 diabetes compared to currently available treatment options. Six subthemes emerged from the data: improved clinical outcomes, reduced mental burden, enhanced sense of normality, reduced burden of significant others, greater societal participation, and lower healthcare costs. Table 2 presents quotations per theme.

**Table 2:** Hoped-for benefits of the BAP for recipients and society.

Sub-themes	Arguments	Quotations
Improved clinical outcomes	• Mitigation of blood glucose fluctuations Prevention of diabetes-related complications • Faster insulin delivery into the bloodstream	*‘’In general, I really hope that we can cure people with type 1 diabetes with a BAP, what we are doing now with the technology is not ideal, which is only providing them lab resources [manufactured insulin] and giving back what they cannot make themselves…Developments like the BAP are exactly the kind of developments what we want for patients… If it would indeed be the case that this [the BAP] will make them insulin independent, and even if that is only for a short period of time, I think there will be a lot of people who will quickly put a side many of their concerns.*’’ − Professional 10
Reduced mental burden	• Reduction of mental effort in self-managing diabetes, allowing more time and freedom to focus on other aspects of life • Emotional relief as individuals no longer need to worry about diabetes-related complications	*‘’Overall, I believe that people with type 1 diabetes want to reduce the time spent managing their disease. Despite the current availability of HCL systems, significant time is still devoted to tasks such as replacing insulin cartridges, monitoring insulin infusion set cannula insertions, and administrating insulin.’’-* Professional 4 *‘’With a BAP, you no longer need to be concerned about your diabetes 24 h a day. It has been researched that a person with diabetes makes between 50 and 400 decisions per day related to their diabetes. So, you can imagine, if all of that is taken care of, how much space you have in your head. No matter how well you manage your diabetes, you can never achieve perfection. We have a list here in the clinic: the 42 factors that influence your blood glucose. I think that there are even more. But of course, you cannot influence all these factors as an individual’’*. – Professional 6 *‘’With a BAP, people with type 1 diabetes are not constantly confronted with it [their disease] …I hope that with a BAP, they [people with type 1 diabetes] are able to occasionally forget about their disease. I believe that these aspects are beneficial for people with type 1 diabetes. That they can entrust the responsibility of disease management to the BAP, allowing them to live as a normal person without having to give it any thought throughout the day.’’ –* Professional 1
Enhanced sense of normality	• The invisibility of the BAP • Reduction of diabetes-related stigma	*‘’A drawback for patients is a treatment that is visible for others, I would therefore suggest that the BAP should be placed where it cannot be seen by others. For instance, when the person [with type 1 diabetes] is walking on the beach. I believe this is very important… the less visible it [the BAP] is, the more one appears to be a person without an illness. If an invisible treatment is feasible, I believe it would be the greatest achievement for some individuals.’’ –* Professional 16
Reduced burden for significant others	• Lower impact of diabetes management on personal relationships • Fewer diabetes incidents affecting significant others, and reducing related fears • Mitigation of guilt and dependency on loved ones	“*Sometimes relationships between children and parents go well, but I also know examples where it went really wrong. One person developed PTSD due to the experience that their parents literally pushed them onto the bed, because the child did not want to administrate insulin, and the parents proceeded to do it themselves.” –* Professional 12 *‘’…partners often wake up when they [persons with type 1 diabetes] experience a hypo at night* …*They notice it if their partner is breathing strangely, or if they [the individual with type 1 diabetes] gets out of bed, then they [the partners] wake up too. I also believe, they [partners] are afraid of low blood sugars, and whether they should call 112. Yes, you never have diabetes alone; you always have it together. Therefore, I think that loved ones would also be very happy and relieved if they [partner with type 1 diabetes] would receive a BAP transplant*.’’ *–* Professional 17
Greater societal participation	• Fewer sick days Enhanced performance at work/school	*‘’So, on the long term, perhaps a BAP transplant might be more advantageous, if people can simply complete their work process until retirement, potentially perform better in school, secure better job opportunities, and consequently contribute more to society*.’’ – Professional 4
Lower healthcare costs	• Improved clinical outcomes through reduced diabetes-related complications • Reduced healthcare consumption • Fewer healthcare professionals needed in diabetes care	*‘’All the time that we are currently spending on explaining and monitoring the devices will no longer be necessary. If we no longer need to do that, or only with a fraction of the people we currently have, then we will need fewer healthcare professionals once the BAP is implemented in clinical care…They [people with type 1 diabetes] will require less care, incur fewer healthcare costs, and will lead healthier lives. This is beneficial for society and for themselves, because they will no longer have type 1 diabetes and will experience a better quality of life*.’’ – Professional 6

#### Improved clinical outcomes

Most professionals hoped that the BAP will mitigate large blood glucose fluctuations in people with type 1 diabetes, leading to fewer hypoglycemic and hyperglycemic events and thereby better long-term clinical outcomes. In addition, several professionals hoped that the BAP will offer faster insulin delivery into the bloodstream, addressing the delay inherent in subcutaneous insulin administration. Furthermore, discussions were often about whether the BAP would be a functional cure; perspectives of the professionals varied. Most professionals hoped that the BAP will functionally cure type 1 diabetes. At the same time, some doubt the potential of BAP to fully cure diabetes. For example, professional 4 said: “*The BAP will never be a cure, because it will not provide glucagon and will not replace the whole pancreas, which has more functions than insulin secretion.’’* Likewise, professional 7 mentioned: “*It will not be a solution for curing diabetes, because then you should solve the T-helper cell attacks.’’* Some professionals anticipated that the BAP may not achieve insulin independence. However, they expected that patients will still be willing to choose the BAP if it means that they need to inject themselves 1–2 times per day. They expected that the burden of injecting once or twice a day could be sufficient to undergo treatment. Also, they anticipated that diabetes-related complications will be prevented with a BAP. If so, they expected that fewer healthcare professionals, such as endocrinologists, nephrologists, and ophthalmologists, will be needed in diabetes care.

#### Reduced mental burden

Professionals often mentioned that managing advanced diabetes technologies, such as HCL systems, still demands considerable mental effort from people with type 1 diabetes, resulting in both cognitive and emotional burdens. These professionals hoped that the BAP would reduce these mental burdens, allowing patients more time and freedom to focus on other aspects of their life*.* Professional 5, for instance, remarked that “*Patients need to constantly think about everything; they are never truly free from their diabetes management’’.* They anticipated that the BAP will simplify self-management, which would be a major benefit, especially for those currently struggling with technology (e.g., ratio calculations). In addition, the majority of professionals highlighted that living with a chronic illness is not just cognitively, but also emotionally exhausting; patients are often worried about potential complications resulting from imperfect diabetes management over time, as well as the occurrence of hypoglycemic events at inconvenient moments. Professionals were hopeful that a BAP transplant will provide emotional relief to individuals with type 1 diabetes.

#### Enhanced sense of normality

The majority of professionals explained that the invisibility of a BAP when it is transplanted into the body could enhance the sense of normality for people with type 1 diabetes. They explained that visibility and audibility (via alarms) of medical devices could drive diabetes stigma, as they could be perceived by others (e.g. employers) as indicators of being ill or vulnerable. In addition, wearable technology can provoke unwanted reactions from others, varying from curiosity to disgust. For example, professional 9 said, *“People with type 1 diabetes do not want to be different from others…so not having people ask: ‘What do you have there?’ or ‘Are you ill*?”.

#### Reduced burden for significant others

Several professionals have pointed out that living with type 1 diabetes can adversely impact social relationships. For example, professional 12 highlighted that relationships between children and parents are often disrupted, particularly in early childhood when the child is unable to manage their condition independently. A BAP could reduce the burden imposed on relationships between parents and their children, as self-management will no longer be necessary. Additionally, several professionals have highlighted that when a hypoglycemic event occurs during sleep, regularly loved ones notice it and must respond. After such an incident, partners frequently fear that it will occur again. Professionals expected that the occurrence of hypoglycemic events will decrease with a BAP. Consequently, those close to individuals with type 1 diabetes may feel less fearful of such events occurring. Patients themselves may experience less guilt toward their partners for not being able to prevent these events, and may feel reduced dependency, potentially enhancing their romantic relationships.

#### Greater societal participation

Some professionals expected that people with a BAP may perform better at work and in school. For instance, professional 4 expected that people with a BAP will take fewer sick days, and will enhance the quality of their performance, as their ability to manage their disease at work will increase. Furthermore, a few professionals envisioned that people with a BAP will perform better in education and will be less likely to drop out, as a significant portion of their cognitive and emotional energy is currently being spent on monitoring their disease rather than on focusing on studies.

#### Lower healthcare costs

Multiple professionals expected that the BAP could positively impact public healthcare expenditure owing to the expected benefits in terms of improved clinical outcomes, reduced healthcare consumption, and the need for fewer professionals working in diabetes care.

### Theme 2: Concerns

Professionals mentioned several concerns regarding the implementation of the BAP in clinical care. Five subthemes were identified: safety and effectiveness, inequitable access, provision of accurate information, control over self-management, and organizational challenges. Table 3 presents quotes for each sub-theme.

**Table 3:** Concerns.

SUB-THEMES	ARGUMENTS	QUOTATIONS
Safety and effectiveness	• Safety concerns for early-phase clinical trial participants • Safety concerns regarding the use of scaffolds, genetically modified cells and pig islets. • Worries about the BAP producing excessive or insufficient insulin • Need for re-transplantation • Concerns about the removability of the BAP in the event of malfunction • The potential requirement for immunosuppressive medication	*‘’If you put it in a scaffold, you can also remove it [the BAP] out of the body. What I am really afraid of, of course, is that the scaffold will leak and that those genetically modified cells can get into the person [with type 1 diabetes], we do not know what will happen then. I mean when genetically modified cells will be used. In practice these cells could become cancer cells, and I think that is a bit scary. You can also imagine that the cancer can grow in the scaffold and grows out of it, right. So yes, then you can say that the layer can protect it. But when it is an aggressive cancer, I can also imagine that it just goes straight through it.’’ −* Professional 12 ‘’*You can always say beforehand: ‘Yes, we don’t know if it works for you… and if it does work, I don’t know how long it will work’… But eventually, they will be confronted with it if it turns out to work for a shorter time than they hoped, or it [the BAP] did not work at all… Yes, that uncertainty, yes, that would be a concern for me. However, it wouldn’t be a reason not to investigate it, let that be clear*.‘’ − Professional 17
Inequitable access	• Concerns about excessive developmental costs, the lack of universal reimbursement policies, and resource scarcity	‘’*I am concerned that people may end up paying for it [the BAP] themselves, and going directly to the company where it is manufactured, once the BAP is likely commercialized. As a result, only those with substantial funds would have access to it. That is something that plays out in healthcare anyway. In the Netherlands, it is less severe than in the United States for instance, but even here in The Netherlands, people who are poor or have a migration background receive worse care simply, because they have less money and fewer resources. The most significant predictor in the Netherlands for good health is high socio-economic status, and that will also come in play with the BAP. You see that with many novel technologies, that nice new interventions actually increase inequality because they are only available to a portion of the population*.’’ – Professional 12 *“If you have everything under control, your diabetes is well managed, you work hard, and it leads to perfect regulation, then you are currently being punished, because you won’t qualify for new treatment, because you are doing too well, that is how it feels for them, I can imagine.’’* − Professional 6
Provision of accurate information	• Concerns about comprehensively explaining all relevant risk • Worries that inflated hopes for a cure may impair individuals’ ability to assess risks and benefits, and anticipate the potential impact of a BAP transplant on their lives	*‘’Then there is another press release in the newspaper about a new therapy successfully transplanted into some mouse, and the next day they [persons with type 1 diabetes] are in my office asking, ‘Did you see that, and when will it be available for people?’’…Well, I think that there is too much hope being offered, like, we are almost there, well definitely not. That is frustrating for people. Like I said, there are people who have hundred thousand euros in the bank for years, to be able to get a transplant. That is quite demotivating for people.*‘’ – Professional 13 “*I tell people in my clinic that they should focus on the [medical device] technology, because that will help them the most in the upcoming years. It will take at least 10 years before diabetes is cured, so do not hope for that.” –* Professional 6
Relinquishing control	• Concerns that sudden loss of control over blood glucose levels could lead to stress for recipients • Worries that excessive reliance on BAP’s functionality may diminish self-management skills over time	“*You undergo the surgery, and afterwards, the glucose levels are consistently normal, they [persons with type 1 diabetes] find that hard to believe because they are very addicted to check their blood glucose levels constantly*.’’ − Professional 8 “*If you are used to doing a lot self-management and suddenly that disappears, then there is a huge emptiness. How will they fill that emptiness? That is really, yes, that is really something. Psychological support is definitely needed.’’ –* Professional 4
Organizational challenges	• Concerns that unclear attributions of responsibilities and ineffective multidisciplinary collaboration across disciplines could hinder implementation	‘’*The conditions and responsibilities must be clearly defined. It may turn out that another team is responsible for the BAP, and perhaps we would not have any role anymore. That is entirely possible. We must prevent people from falling through the cracks, because their diabetes is resolved, ensuring they continue to be monitored in some way. We must ensure they don’t disappear from view.’’* – Professional 4 *‘’I am concerned whether people [with a BAP] can act accurately in case of an emergency. What if something goes wrong [with the BAP] and the sugar becomes disrupted? You want to prevent them [ individuals with a BAP] from ending up in the ICU with a ketoacidosis. They need to be able to recognize signals, and we need to continue repeating and educating them that.*’’ – Professional 16

#### Safety and effectiveness

The primary concerns centered on the safety of the BAP for transplantation into early phase clinical trial participants, especially when alternative treatment options exist. Professionals envisioned challenges for researchers to evaluate the safety and efficacy of a complex product, such as the BAP, generated from cells derived from multiple sources. Specifically, worries were voiced about the safety of using scaffolds, genetically modified cells, and pig islets to generate the therapy, with concerns about potential (long-term) adverse events such as scaffold leakage, tumorigenicity, and zoonosis. They anticipated that adverse events, especially in young children, could result in major delays in implementation of the BAP. Conversely, most professionals were not worried about the risks associated with the prospective surgical procedure, anticipating the BAP to be a small, subcutaneously inserted product and the procedure to require minimal anesthesia. Aside from safety concerns, some professionals doubted the effectiveness of the BAP, even with accurate testing in clinical trials, fearing it might produce excessive or insufficient insulin, potentially leading to hypo- or hyperglycemic events. Additionally, many professionals foresaw the need for recipients to eventually undergo another transplantation procedure. Two professionals proposed the development of a system in which beta cells can be easily and periodically reinjected when they malfunction, such as with a port-a-cath-like device. Also, concerns have been raised about the removability of the BAP in the event of malfunction. Finally, most professionals indicated that they would offer the BAP solely to individuals with type 1 diabetes if it proved safe and effective without requiring immunosuppressive medication, as they believed the benefits of the BAP would not outweigh the risks related to the medication.

#### Inequitable access

Professionals voiced concerns about the future accessibility of the BAP for the majority of individuals with type 1 diabetes, citing reasons such as (1) excessive developmental costs, (2) reimbursement policies, and (3) resource scarcity. First, most professionals foresaw that the BAP will be costly to develop. Several professionals have emphasized the importance of considering cost-effectiveness when clinically translating potentially expensive therapies, such as the BAP, to ensure that healthcare remains affordable. In addition, they expected that reaching a sufficient production volume to lower costs and increase accessibility will either take a long time or will never be achieved. Hence, some expressed worries that only wealthy individuals with type 1 diabetes will have the means to afford a costly BAP in the future, contributing to growing inequality. Second, some professionals expressed concerns regarding the absence of universal reimbursement for the BAP, akin to novel technologies such as continuous glucose monitoring (CGM) devices. They expected that reimbursement for the BAP may only be extended to those for whom it represents a last resort, and not to individuals who are able to effectively manage their condition. Therefore, some professionals suggested that the BAP should be available for those groups who are currently excluded from making use of novel technologies such as CGM devices. Third, a few professionals mentioned that the shortage of deceased donors (potentially needed as a source of cells) could be a barrier limiting the availability of the BAP. They suggested that researchers should therefore prioritize developing insulin-secreting cells derived from stem cells to address this limitation. Furthermore, some professionals expressed worries that access to the BAP for individuals with type 1 diabetes might be restricted to specialized centers in metropolitan areas, rather than being widely available across the country. They stated that this could result in lengthy waiting lists and limited access to people living in rural areas.

#### Provision of accurate information

Professionals reported various topics that must be addressed in the informed consent process to ensure accurate information about the BAP for individuals with type 1 diabetes, as summarized in Table 4**.** First, numerous professionals expected challenges in comprehensively explaining all relevant risks owing to the complex nature of the BAP. They specifically referred to the various biological materials involved, which makes the risk assessment complex because each component presents its own set of risks and ethical considerations. Given that decision-making relies on carefully weighing risks and benefits, some professionals have raised concerns about the validity of informed consent when the information on risks is too complex. They thought that when informing individuals about the BAP, identifying individual information needs and appropriately tailoring information would be particularly essential. Second, some professionals noted that inflated hopes for a cure might affect individuals’ ability to assess risks and benefits, and realistically anticipate the potential impact of a BAP transplant on their lives. They were worried that people with type 1 diabetes may experience disappointment if —potentially unrealistic—expected outcomes do not occur. In relation to this, many professionals emphasized the need for cautious and transparent media reports on the development of the BAP so as not to create unrealistic expectations. Professionals have suggested that reports should include accurate information about when and for whom the BAP might be a suitable treatment option.

**Table 4:** Topics to be addressed in the informed consent process based on professional’s perspectives.

Efficacy	- The chance of success (%), and the expected type of outcomes (e.g. lifelong insulin independence, reducation in hypoglycemia/hyperglycemia events, decrease in diabetes-related complications and self-management burden) - When the BAP will start working
Risk Assessment	Standardized information - Whether the potential benefits outweigh the risks - Comparison to alternative available treatments, including risks, benefits and implications for daily life - Surgical risks - Potential adverse events - Risk associated with immunosuppression, if necessary, which is drug-specific and generic (such as an increased risk of certain cancers, infections etc.) - Retrievability of the BAP (i.e. whether it could be removed) - Risks of disease recurrence and graft failure - Risk of product leakage or rupture Personalized information - Impact of treatment on lifestyle and quality of life Individual and familial needs (e.g. impact on care-givers)
Safety	- Clinical trials study results
Transplant procedure	- Average duration of hospitalization Surgical invasiveness - Procedural complexity - Anesthesia necessity Wound healing
Size and location	- Size - Visibility - Transplantation location - Accessibility of the BAP in order to check its graft functioning - Durability - Impact of size and location on daily life
(Biological) materials used	- Source of cells (deceases donors, animals and/or genetically modified cells) - Risks associated with the material being used (e.g. zoonoses or tumorigenesis)

#### Control over self-management

Many professionals have expressed concerns that individuals with type 1 diabetes may face difficulties relinquishing control over their disease management after undergoing transplantation. They expected that the abrupt loss of control over blood glucose levels require a mental transition that could lead to considerable stress among recipients. Professional 4,5, and 8 proposed offering psychological support and providing a CGM device to recipients who struggle with relinquishing control over their disease management. They anticipated that during the early stages following transplantation, additional guidance and insight into blood glucose levels would help recipients build trust and familiarity with the BAP. At the same time, they also advocated gradually reducing recipients’ reliance on CGM devices over time. In addition, some professionals were concerned that when individuals with a BAP rely too heavily on the functionality of the therapy and relinquish control, their self-management skills may deteriorate over time, which might ultimately pose new health threats, when the technology starts to fail. They highlighted the importance of educating individuals during consultations on self-management, for example, on lifestyle aspects, recognizing warning signs to prevent ketoacidosis.

#### Organizational challenges

Shortly after transplantation, professionals anticipated that intensive monitoring will be required for individuals with BAP transplants. Once the safety and effectiveness of the BAP are confirmed in recipients, most professionals expect a decrease in the level of care required for these individuals. However, multiple professionals expected that unclear attributions of responsibilities and ineffective multidisciplinary collaboration involving transplant surgeons, endocrinologists, manufacturers, patient associations, and regulatory bodies could be barriers to successful BAP implementation. For example, in the event of BAP malfunctioning, concerns have been raised about whether the responsibility to provide support to patients lies with professionals or manufacturers. In addition, they emphasized the necessity of sharing experiences, data, and knowledge to prevent adverse events and avoid unnecessary duplication of efforts across clinics.

### Theme 3: Allocating the BAP during initial implementation

Most professionals believed that the BAP could benefit all people with type 1 diabetes. These professionals hesitated to prioritize specific target groups in case of limited government funding or healthcare insurance, hindering access to the BAP. The professionals had diverse views on who would be prioritized to receive the BAP. In total, they mentioned four target groups that they deemed most in need of the BAP. Table 5 summarizes the arguments and presents the quotes for each suggested target group.

**Table 5:** Allocating the BAP during initial implementation.

Early Implementation		
Considerations	Arguments	Quotations
**Lacking effective treatment options**	- Persons without effective treatment are most likely to experience improvements in short-term health outcomes	‘’ *I find it logical to transplant it in poorly regulated patients who incur high healthcare costs and also have a poor quality of life. You may choose this group where things are going very poorly. Because they would benefit the most from such treatment’’*. − Professional 6
**Diabetes-related mental health issues**	- Persons with diabetes-related mental health issues are most likely to experience improvements in quality of life	*‘’ The group of people who prefer to ignore their diabetes, because currently with novel HCL systems you [people with type 1 diabetes] do need to invest in it and spend time on it. You [people with type 1 diabetes] really need to set it up, adjust an and calibrate it, which is often necessary, especially in the beginning. There is a group of people who prefer to act as if they don’t have diabetes. They inject long-term insulin, and ignore it further. Yes, those people prefer not to think about their diabetes, they do not want to constantly deal with a device. And sometimes they do not like the visibility of the device on their body… they just prefer to stick something on and then not think about their diabetes for two weeks. And in my opinion, the BAP would be ideal for that group. It is not a small group at all, but they are currently really underserved’’. −* Professional 12 *‘’ I think, that the BAP should go too people who have a lot of mental problems, psychologically, because of their diabetes. I think they are just mentally burdened so much, that is actually, the largest group of patients who would benefit from that [the BAP]’’. −* Professional 14
**Vulnerable persons who require caregiver support**	- To relieve healthcare professionals and parents	‘’ *When you have people [with type 1 diabetes] who have a HCL system, and are 80 or so and can’t handle it themselves, they go to a nursing home. Should they then go back to using an insulin pen, with a terrible blood glucose level, or can you provide them a BAP? That is actually quite an interesting question, that nobody [healthcare providers] has to pay attention to their diabetes anymore*… *They cannot manage their diabetes, and then you really relieve them, both the caregivers and the people [with type 1 diabetes] themselves’’.* − Professional 4 ‘’*I always hope for the children, I am honest about it, because if you treat the children well, the parents also have their life’s back. Because, of course, a whole family system resolves around it [type 1 diabetes]. So, then you have happy families again, that would be wonderful. What is left [in number of BAPs] can be used for the adult world.’’ −* Professional 7
**Age**	- Younger persons can benefit longer - Long-term complications can be prevented - The preservation of endogenous insulin production can be prolonged	‘’ *I think if you give young people a BAP, it ensures that they are well regulated from the start of life with the best technology we have. Consequently, long-term complications, quality of life, labor participation and participation in the society will be significantly be improved till the end of life’. −* Professional 17 *‘’.. as early as possible, because with diabetes we know that the earlier you treat people well, the longer they also maintain their own insulin production. So, there are studies where they tightly regulate and not tightly regulate people with type 1 diabetes. And people who are tightly regulated maintain their own insulin production longer, because their cells get some rest.’’ − Professional 12* ‘’*I find it difficult to transplant the BAP in children, especially when they are between twelve and sixteen, to expose them to sometimes parental pressure. You know, ‘you should try this’, I think you should wait until they can make a choice for themselves. Because, it is a novel technology you know, there are always drawback to it.*’’ − Professional 8

#### Lacking effective treatment options

Many professionals advocate prioritizing individuals with type 1 diabetes who currently experience poor regulation owing to the lack of an effective treatment option, such as athletes, those with highly fluctuating glucose levels, individuals who are allergic to device adhesives, or those struggling with advanced devices owing to insufficient knowledge or skills. They reasoned that a BAP transplant could yield the greatest short-term health impact in these target groups and expected significant improvements in diabetes-related outcomes in these individuals.

#### Diabetes-related mental health issues

Multiple professionals have suggested prioritizing individuals with type 1 diabetes who experience the highest mental burden associated with the condition, including those who tend to deny their disease. They argued that this specific group personally gained the most from a BAP as it could substantially enhance their quality of life.

#### Vulnerable people who require caregiver support

Some professionals proposed prioritizing vulnerable groups to try to alleviate societal burdens, such as providing the BAP to individuals living in nursing homes or to children to ease parental caregiving responsibilities. They believed that prioritizing these individuals could have a significant societal impact, allowing caregivers to participate more fully in society without the burden of managing diabetes, in addition to benefitting the individual.

#### Age

Most professionals highlighted the benefits of selecting young individuals, stating that these individuals have their entire lives ahead of them. Prioritizing these groups for BAP treatment may lead to greater long-term health benefits, improved quality of life, and the prevention of (long-term) complications. However, some professionals anticipated that developing the BAP will be less relevant as device-based technologies advance, because most long-term complications will be prevented due to better regulation of glucose levels.

## Discussion

This interview study aimed to explore diabetes healthcare professionals’ perspectives on the potential future clinical implementation of the BAP for persons with type 1 diabetes.

This study found that professionals envisioned that a BAP transplant could bring psychological and/or social relief, such as ‘*reducing mental burden’* for recipients and their significant others, alongside medical benefits (e.g. improved glycemic control). Thus, providing psychological support post-transplantation will be crucial, as professionals expect that people with type 1 diabetes may struggle to adapt to ‘*relinquishing control’* over self-management. Both professionals and individuals with type 1 diabetes expected that a BAP transplant could offer benefits across medical, psychological, and social domains, and they shared concerns about the safety of the biological material used, long-term efficacy of the BAP, relinquishing control, and inequitable access [10]. Additionally, prioritizing younger patients in the initial stages of implementation was also suggested by people with type 1 diabetes [10]. Compared with individuals with type 1 diabetes, professionals foresee societal benefits for people undergoing BAP transplantation, such as greater ‘*societal participation*’ and ‘*lowering healthcare costs’*. These results can be explained by studies showing that individuals with type 1 diabetes often have lower levels of performance at work and school compared to people without a chronic illness, mainly due to lower energy levels and diabetes-related complications [29], [30], [31]. Therefore, improved illness management through the BAP may lead to higher productivity. Additionally, the total healthcare expenditure for people with diabetes in the Netherlands are high, with most hospital costs seemingly caused by diabetes secondary complications [32], [33]. These findings are similar to studies performed in other European countries [34], [35]. BAP is believed to reduce diabetes-related complications in recipients, and thus improve social participation and lower healthcare costs. Furthermore, while professionals raised concerns about ‘*organizational challenges*,’ individuals with type 1 diabetes mostly worry about the potential intrusiveness of the follow-up procedure [10]. These divergent findings suggest that diabetes healthcare professionals tend to consider the potential impact and effects of innovative therapies, such as the BAP, on healthcare and society, while individuals, understandably, primarily focus on the personal benefits and drawbacks that a new therapy might offer.

Professionals anticipated that the BAP could offer medical benefits beyond insulin independence for individuals with type 1 diabetes. For instance, improved blood glucose stability could be particularly beneficial for those experiencing fear of recurrent episodes of hypoglycemia or those struggling with hypoglycemia unawareness [36]. Severe hypoglycemic events and the fear of these events have a significant impact on the quality of life of people with type 1 diabetes [37], [38]. Therefore, if BAP transplants can effectively reduce severe hypoglycemic and hyperglycemic events, the benefit of maintaining stable blood glucose levels may outweigh the need for daily insulin injections. This aligns with the ongoing debate on islet transplantation that insulin independence may not be the sole indicator of success^34^. Multiple studies have shown improved long-term health-related quality of life (HRQOL) in people with type 1 diabetes after islet transplantation, compared to the pre-transplant situation, even if they do not achieve insulin independence [36], [39], [40], [41]. Notably, Foster et al. found significant reductions in both diabetes-related distress and fear of hypoglycemia post-transplantation, with no statistical difference in HRQOL scores between insulin-independent and insulin-dependent participants[41]. Hence, the development of the BAP should continue even if complete insulin independence is not achieved in all patients.

At a societal level, this study showed that professionals are concerned about ensuring equitable access to the BAP. These concerns are neither novel within the realm of type 1 diabetes treatment nor unique to regenerative medicine therapies [42]. At present, the primary barrier for people with type 1 diabetes to accessing advanced technological devices lies in the lack of coverage or insurance within healthcare systems due to restrictive eligibility criteria [16]. With regard to the BAP, professionals foresee an even greater challenge in ensuring accessibility. The tissue engineering technologies necessary for developing cell-based therapies require specialized personnel and laboratory resources. These highly specialized facilities are particularly lacking in less-developed countries. Therefore, it is imperative to contemplate accessibility of care early in the development process. Moving forward, research groups, grant providers, academic institutions, and manufacturers should make an effort to design and develop a BAP in a manner that ensures affordability and accessibility so that most individuals with type 1 diabetes worldwide can benefit from this therapy. To achieve this, adequate and fair reimbursement policies must be established, and international collaboration to share scientific knowledge must be encouraged [42].

Finally, this study provided the insight that professionals hold diverse perspectives, including moral perspectives, on prioritization, emphasizing the need for further dialogue to eventually develop clinical guidelines to assist diabetes care professionals in allocating the BAP during the initial implementation phase. We observed that healthcare professionals adhere to different resource allocation principles: some focus on the interests of individuals (e.g., patients for whom no effective treatment is available), while others focus on those of society (e.g., the need for caregiver support). Thus, professionals are considering not only what is just and fair for the individual patient, but also what could be consequently beneficial to society or the patient group as a whole to achieve the greatest overall good. Furthermore, these considerations could be linked to various distributive justice theories, such as like egalitarianism and utilitarianism [43]. From an egalitarian perspective, one could argue for prioritizing patients for whom no effective treatment is currently available, whereas from a utilitarian perspective, one might prioritize vulnerable patients or children. Additional conceptual research into these various accounts, within the context of allocating the BAP during early implementation stages, may offer valuable insights that can be used in the development of ethically responsible clinical guidelines for BAP.

### Strengths and limitations

Various strengths of the study are noteworthy. First, to the best of our knowledge, this is the first study on professionals’ perspectives regarding the BAP. The perspectives of professionals on novel technologies have often been neglected in research, despite their important role as gatekeepers to implementation in the clinic [13]. Second, the involvement of experts in medicine, ethics and psychology in the research team enriched the analysis and interpretation of findings, ensuring a holistic understanding of implementing BAPs in clinical care. Third, employing a qualitative approach stimulated in-depth discussions with respondents about this hypothetical therapy. This provided a comprehensive and rich overview of their perspectives on potential implications of the BAP for diabetes care in the future. Last, by purposefully sampling selected professionals with diverse professional backgrounds, we were able to include a diverse and multidisciplinary sample from varying geographical areas and hospitals. This diversity enhances the generalizability of the study’s findings.

A number of limitations affects the interpretation of the findings. First, a challenge encountered in conducting this research was to inform professionals in sufficient and adequate technical detail about a hypothetical product without unduly influencing their viewpoints. We addressed this by providing a standardized PowerPoint presentation about the BAP prior to the interview. Second, the study was conducted in the context of the Netherlands, which potentially limits generalization of findings to other countries, with different healthcare systems and reimbursement policies. Conducting an international comparative study would offer a broader understanding of perspectives regarding the BAP. Lastly, the clinical expertise and differing approaches to risk and benefits among diabetes healthcare professionals (i.e. nurses, endocrinologists or a pancreas transplant surgeons), may have introduced bias or shaped reported perspectives on the potential of the BAP for treating persons with type 1 diabetes. For instance, nurses may have a more holistic view of the day-to-day impact of diabetes management as they might spend more time in discussing self-management and psychosocial functioning with persons with type 1 diabetes, while endocrinologists might focus more on the potential clinical efficacy of the BAP. Surgeons, on the other hand, might be more concerned with procedural risks. However, having included multiple diabetes professions in this study, we were able to provide a more comprehensive understanding of the potential of the BAP.

### Future research

Looking ahead, given the results on the potential psychological and social benefits the BAP could offer recipients, researchers must go beyond evaluating just clinical outcomes post-transplantation in order to assess its overall effectiveness. For instance, comparing HRQOL scores of persons with type 1 diabetes before and after transplantation can reveal the impact of the BAP on their daily lives. These scores could also guide clinicians and policy-makers on the need for ongoing support and interventions to improve HRQOL post-transplantation. Additionally, conducting a comparative study among sub-groups (e.g. across professions) might be relevant before implementing the BAP in clinical care to identify specific concerns per profession. We propose employing a mixed-methods approach, including the use of a survey to allow for quantitative comparisons between sub-groups and evaluation of frequency of themes. The results of our study, including the identified themes and sub-themes, could serve as a groundwork for such a mix-methods study. Finally, whether the BAP is beneficial for people with type 1 diabetes is directly influenced by alternative treatment options and other innovations in diabetes treatment that are being developed in parallel. Therefore, in the future, it may be necessary to repeat this study or reconsider the suggested considerations by professionals as alternative treatments evolve.

## CRediT authorship contribution statement

**Dide de Jongh:** Formal analysis, Writing – original draft, Methodology, Investigation, Formal analysis, Conceptualization. **Eline Bunnik:** Writing – review & editing, Supervision, Conceptualization. **Ozcan Behiye:** Writing – review & editing. **Robert Zietse:** Writing – review & editing. **Emma Massey:** Writing – review & editing, Supervision, Project administration, Conceptualization.

## Funding

This project has received funding from the European Union’s Horizon 2020 research and innovation programme under grant agreement no. 874700.

## Declaration of competing interest

The authors declare the following financial interests/personal relationships which may be considered as potential competing interests: Dide de Jongh reports financial support was provided by European Commission. If there are other authors, they declare that they have no known competing financial interests or personal relationships that could have appeared to influence the work reported in this paper.
