# Supplementary material for: The bio-artificial pancreas to treat type 1 diabetes: Perspectives from healthcare professionals in the Netherlands
Source: J Clin Transl Endocrinol. 2024 Oct 17;38:100372. doi: 10.1016/j.jcte.2024.100372 (PMC11536005; doi:10.1016/j.jcte.2024.100372)
Supplement: Supplementary Data 1 [file mmc1.docx]

## Supplement 1

### Interview guide (translated)

Topic 1: Challenges in current diabetes care

- What do you find most challenging about your work as a caregiver for people with type 1 diabetes?
- What would make your work easier?

Topic 2: Needs regarding innovative treatment options

- In your opinion, how does having type 1 diabetes affect peoples’ daily lives?
- What are the needs and wishes of people with type 1 diabetes for novel treatment options?

Topic 3: Developments in current treatment options

- What are the greatest benefits and limitations of the current technological devices used to monitor blood glucose levels and administer insulin?
- What are the greatest benefits and limitations of pancreas or islet cell transplantation treatments for persons with type 1 diabetes?

Topic 4: Expectations on the bio-artificial pancreas as a treatment option

- How could the bio-artificial pancreas impact daily lives of people with type 1 diabetes?
- What are in your view potential advantages and disadvantages of the bio-artificial pancreas compared to the current alternative treatment options for people with type 1 diabetes?

Topic 5: Considerations regarding target group selection

- When the bio-artificial pancreas is implemented in clinical practice, which diabetes target group should the prioritized for its allocation first, and why?
- What is your perspective on the target group that should be considered for early-phase bio-artificial pancreas studies?

Topic 6: Considerations regarding transplantation

- What would be the preferred location for transplanting the bio-artificial pancreas in the human body, and what are your considerations?

Topic 7: Obstacles and facilitators in the implementation of the bio-artificial pancreas

- What is your view on potential obstacles when the bio-artificial pancreas is implemented in diabetes care?
- What requirements should the bio-artificial pancreas meet before you would recommend this potential treatment to your patients?

Topic 8: Attitude towards genetically modified, porcine, and donor cells

- The product may contain cells from deceased donors. Can you tell me how you feel about that?
- The bio-artificial pancreas may contain insulin-producing cells from pigs. Can you tell me how you feel about that?
- What are your views on using genetically modified cells to create a transplantable bio-artificial pancreas?

Topic 9: Attitude towards pre-transplantation information provision

- What are in your view the greatest challenges regarding information provision about the bio-artificial pancreas?
- Do you have a suggestion to improve information provision regarding the

bio-artificial pancreas as a potential treatment option?

Topic 10: Attitude regarding post-transplantation monitoring

- What factors are important to enable the monitoring of people with a bio-artificial pancreas post-transplantation?

Topic 11: Future perspective

- When will the bio-artificial pancreas be a successful treatment option for people with type 1 diabetes?
- How do you think diabetes care and facilities will change when bio-artificial pancreases are widely accessible for people with type 1 diabetes?

### Interview guide in Dutch

Topic 1: Uitdagingen in de huidige diabetes zorg

- Wat vindt u het meest uitdagend aan uw werk als zorgverlener rondom de behandeling van mensen met diabetes type 1?
- Wat zou uw werk op dit moment makkelijker maken?

Topic 2: Behoeften rondom innovatieve behandelopties

- Wat voor invloed heeft het hebben van diabetes type 1 volgens u op het dagelijkse leven van patiënten?
- Wat zijn de behoeften en wensen van mensen met diabeten type 1 met betrekking tot innovatieve behandelopties?

Topic 3: Ontwikkelingen rondom huidige behandelopties

- Wat zijn volgens u de baten en beperkingen van de huidige technologische apparaten om bloedglucose levels te monitoren en insuline toe te dienen?
- Wat zijn volgens u de baten en beperkingen van een alvleesklier of eilandjes van Langerhans transplantatie behandeling voor patiënten?

Topic 4: Verwachtingen rondom de bio-artificiële alvleesklier als behandeloptie

- Op welke manier zou de bio-artificiële alvleesklier het dagelijkse leven van uw patiënten kunnen beïnvloeden?
- Wat zijn volgens u de verwachte voor - en nadelen van de bio-artificiële alvleesklier in vergelijking met beschikbare alternatieve behandelopties voor personen met diabetes type 1?

Topic 5: Overwegingen rondom het selecteren van een doelgroep

- Wanneer bio-artificiële alvleesklieren zijn geïmplementeerd in de klinische praktijk, aan welke diabetes doelgroep zou u deze behandeling dan als eerste aanbevelen, waarom?
- Welke diabetes doelgroep zou voor vroege fase bio-artificiële alvleesklier studies in aanmerking kunnen komen? Wat zijn uw overwegingen?

Topic 6: Overwegingen rondom transplantatie

- Welke plek zou uw voorkeur hebben voor het transplanteren van een bio-artificiële alvleesklier, en wat zijn uw overwegingen?

Topic 7: Obstakels en facilitators rondom de implementatie van de bio-artificiële alvleesklier

- Wat ziet u als mogelijke obstakels wanneer de bio-artificiële alvleesklier wordt geïmplementeerd in de diabeteszorg?
- Aan welke eisen zou de bio-artificiële alvleesklier moeten voldoen voordat u deze behandeling zou voorstellen aan uw patiënten met diabetes type 1?

Topic 8: Attitude rondom genetisch gemodificeerde, varkens en donor cellen

- Het kan zijn dat er cellen van overleden donoren in het product zitten. Kunt u mij vertellen hoe u daar tegenover staat?
- Mogelijk zijn de insuline-producerende cellen in de bio-artificiële alvleesklier afkomstig van varkens. Kunt u mij vertellen hoe u daar tegenover staat?
- Wat zijn uw opvattingen over het gebruiken van genetisch gemodificeerde cellen om een transplanteerbare bio-artificiële alvleesklier te maken?

Topic 9: Attitude rondom informatievoorziening pre-transplantatie

- Wat zijn volgens u de grootste uitdagingen met betrekking tot de informatievoorziening over de bio-artificiële alvleesklier?
- Heeft u een suggestie voor het verbeteren van de informatievoorziening voor patiënten over een mogelijke behandeling met een bio-artificiële alvleesklier?

Topic 10: Attitude regarding monitoring post-transplantation

- Welke zaken zijn van belang bij het monitoren van patiënten met een bio-artificieel alvleesklier post-transplantatie?

Topic 11: Toekomst perspectief

- Wanneer is de bio-artificiële alvleesklier volgens u een succesvolle behandeloptie voor mensen met diabetes type 1?
- Op welke manier denkt u dat de diabeteszorg gaat veranderen als bio-artificiële alvleesklieren breed beschikbaar gaan worden voor mensen met diabetes type 1?

## Supplement 2

### Script of the presentation (translated)

At this moment, various researchers working in the field of regenerative medicine are developing a vascularized, immune-protected, transplantable bio-artificial pancreas for people with type 1 diabetes. The development of this product is still in its infancy, and is not expected to be available soon. What does a bio-artificial pancreas consist of? The research approach is that the pancreas will be entirely made of biological material and consists of different types of cells derived from multiple sources. It will probably contain patients own cells, making the product personalized. In addition, genetically modified cells from donated placentas may be used. These cells are added to the product to support vascularization and prevent an immune reaction in the transplanted person. The Islets of Langerhans should ensure that the product produces sufficient insulin. The islets may be derived from deceased donors, pigs or induced pluripotent stem cells. There is a shortage of deceased donors, hence researchers are evaluating whether pig islets or induced pluripotent stem cells can be used to provide sufficient insulin secretion. The cells derived from multiple sources will be mixed and protected by a scaffold, for example a so-called hydrogel. The researchers aim to ensure that once the bio-artificial pancreas is transplanted into a person with type 1 diabetes, the individual will no longer need to self-administer insulin, as they will hopefully become insulin-independent. The bio-artificial pancreas is immune-protected, so recipients will not need to take immunosuppressive medication. In summary, this interview focusses on the development of a vascularized, immune-protected bio-artificial pancreas made entirely of biological material, which researchers intend to transplant into patients with type 1 diabetes.

Why do we conduct this interview study? To investigate the expectations of diabetes professionals regarding the development of the bio-artificial pancreas for people with type 1 diabetes. Diabetes professionals are relevant stakeholders because of their gatekeeping role in the clinic. What do they expect as possible barriers and facilitators, and which persons should be prioritized first, and why? By evaluating the perspectives of diabetes professionals regarding the clinical development of the bio-artificial pancreas early on, it will be possible to incorporate their viewpoints into further development of this potential future therapy. For example, researchers do not yet know where the product will be placed in the body. What could be a suitable implementation site? Does this potentially innovative treatment offer a suitable alternative compared to the treatment currently available for people with type 1 diabetes? What are the risks diabetes professionals worry about when the bio-artificial pancreas will be transplanted into a patient’s body? These are all open questions I am going to ask you in the next half hour.

### Script in Dutch

Op dit moment zijn verschillende onderzoeksgroepen bezig met de ontwikkeling van een gevasculariseerde, afweer-beschermende, transplanteerbare bio-artificiële alvleesklier voor personen met diabetes type 1. De ontwikkeling van dit product staat nog in de kinderschoenen, dus de therapie waarover ik het ga hebben is nog toekomstmuziek. Waar bestaat een bio-artificiële alvleesklier uit? Onderzoekers beogen om de alvleesklier volledig uit biologisch materiaal te maken. De cellen die de onderzoekers willen gebruiken en mixen om een functionele therapie te kunnen maken, zijn afkomstig uit meerdere bronnen. Er worden wellicht patiënt eigen cellen gebruikt, waardoor de therapie gepersonaliseerd kan worden. Daarnaast worden er misschien cellen gebruikt die genetische gemodificeerd zijn afkomstig van gedoneerde placenta’s. Deze cellen kunnen aan het product worden toegevoegd zodat er bloed toevoer kan ontstaan en er geen immuunreactie kan optreden bij de getransplanteerde persoon. En, de eilandjes van Langermans in het product moeten ervoor zorgen dat het product voldoende insuline gaat produceren. De eilandjes zullen waarschijnlijk afkomstig zijn van overleden donoren, varkens of wellicht van geïnduceerde pluripotente stamcellen. Er is een tekort aan overleden donoren waardoor er op dit moment ook onderzocht wordt of er gebruik kan worden gemaakt van varkens eilandjes of geïnduceerde pluripotente stamcellen die insuline kunnen produceren. Een *scaffold*, bijvoorbeeld een hydrogel, wordt ingezet om de cellen die de bio-artificiële alvleesklier vormen te beschermen in het lichaam. Het doel van de onderzoekers is uiteindelijk dat wanneer de alvleesklier is getransplanteerd in een persoon met diabetes type 1, deze persoon zelf geen insuline meer hoeft toe te dienen. De bio-artificiële alvleesklier is afweer-beschermend waardoor getransplanteerde personen geen toxische immunosuppressiva medicatie hoeven te slikken. Samengevat, het interview gaat dus over de ontwikkeling van een gevasculariseerde, afweer-beschermde bio-artificiële alvleesklier die onderzoekers willen transplanteren in mensen met diabeten type 1, en volledig bestaat uit biologisch materiaal.

Waarom voeren we deze interview studie uit? Om alvast de verwachtingen van diabetes professionals te onderzoeken rondom te ontwikkeling van de bio-artificiële alvleesklier voor patiënten met diabetes type 1. Diabetes professionals zijn een relevante stakeholder door hun poortwachters rol in de kliniek. Wat zien zij als mogelijke barrières en facilitators, en welke patiënten moeten volgens hen als eerste in aanmerkingen komen voor deze therapie, en waarom? Door het perspectief van diabetes professionals tegenover de ontwikkeling van een bio-artificiële alvleesklier nu al te onderzoeken, kan hun perspectief worden meegenomen in de verdere ontwikkeling van deze mogelijke toekomstige behandeling. De onderzoekers weten bijvoorbeeld nog niet waar het product in het lichaam wordt geplaatst. Wat kan een mogelijke geschikte implementatie plek zijn? Biedt deze mogelijke innovatieve behandeling een geschikt alternatief in vergelijking met de behandelingen die nu beschikbaar zijn voor mensen met diabetes type 1? Over welke risico’s maken diabetes professionals zich zorgen wanneer de bio-artificiële alvleesklier is getransplanteerd in het lichaam van een persoon met diabetes type 1? Dit zijn allemaal vragen die ik u het komende half uur ga stellen.
